# Responses of rumen microorganisms and metabolites to different roughage of domesticated Tibetan sheep

**DOI:** 10.3389/fmicb.2023.1247609

**Published:** 2023-08-17

**Authors:** Yue Ren, Yangzhong Zhaxi, Renzeng Ciwang, Zhengwen Wang, Mengjun Liu

**Affiliations:** ^1^Institute of Livestock Research, Tibet Academy of Agricultural and Animal Husbandry Sciences, Lhasa, China; ^2^Key Laboratory of Animal Genetics and Breeding on Tibetan Plateau, Ministry of Agriculture and Rural Affairs, Lhasa, China; ^3^Key Laboratory of Grassland Ecosystem, College of Grassland Science, Gansu Agricultural University, Lanzhou, China

**Keywords:** Tibetan sheep, wheat straw, whole corn silage, rumen microorganism, rumen metabolites

## Abstract

Tibetan sheep can utilize high fiber feeds well. However, the mechanisms of rumen microbiota and metabolites in response to different roughage in a housed environment are still unclear. We fed Tibetan sheep with three different roughage diets: 50% whole corn silage (TS), 50% wheatgrass group (TW), and 25% each of whole corn silage and wheatgrass (TM). Subsequently, meat traits, rumen contents 16S rRNA and metabolomics were studied. The results showed that feeding wheat straw to Tibetan sheep significantly increased the abundance of bacteria such as *Ruminococcus* and *Succiniclasticum* in the rumen. These microorganisms significantly increased metabolites such as beta-alanyl-L-lysine, butanoic acid and prostaglandin E2. Eventually, production performance, such as carcass weight and intramuscular fat and meat quality characteristics, such as color and tenderness were improved by altering the rumen’s amino acid, lipid and carbohydrate metabolism. This study demonstrated that including 25% wheatgrass and 25% whole corn silage in the diet improved the performance of Tibetan sheep, revealing the effect of the diet on the performance of Tibetan sheep through rumen microorganisms and metabolites.

## Introduction

Tibetan sheep are the most dominant animal in the alpine meadows of the Qinghai-Tibetan Plateau (QTP), providing local herders with meat, milk, wool and other primary means of production and livelihood. However, the pattern, process and function of alpine grassland ecosystems and animal husbandry have changed in recent years due to the overgrazing of grasslands, resulting in ecological and environmental problems such as grassland degradation and desertification (Lu et al., 2017). The fundamental contradiction of the grassland ecosystem is that the supply of grasslands in the cold season cannot meet the nutritional needs of Tibetan sheep, and overgrazing is still insufficient to feed them (Qi et al., 2017).

Intensive housing or supplemental feeding of Tibetan sheep in the cold season is a feasible option to protect the grassland ecological environment and improve the productivity of Tibetan sheep in the cold season. Many studies have found that housing or supplementing Tibetan sheep with different protein and energy levels in the cold season can effectively improve the performance of Tibetan sheep (da Silva et al., 2020). In addition, with the improvement of people’s life and consumption level, people pay more attention to the quality of mutton, such as tenderness and color. Numerous studies have confirmed that different diets can regulate the rumen fermentation parameters and microflora composition and metabolite accumulation (Xue et al., 2017; Fernandez-Turren et al., 2020), which directly or indirectly affects sheep growth performance, carcass characteristics and meat quality of sheep (Wang X. et al., 2020; Zhang et al., 2022a). Whole plant corn silage is widely used as a high quality roughage for ruminants, providing them with a large number of nutrients and digestible fiber (Guo et al., 2022); Wheat straw has the characteristics of high yield and roughage resistance, but due to the high fiber content, often can not be fed too much to cattle and sheep (Mahmoudi-Abyane et al., 2020). A previous study found that corn silage was more valuable for beef cattle than added wheat straw (Zhang H. et al., 2022), and research showed that the sheep had lower digestibility of wheat straw, while camels had higher digestibility when fed wheat straw (Khattab et al., 2021). This may indicate that animals with vital rumen function have better digestion of high fiber roughage. Tibetan sheep have formed a rumen flora that is more likely to decompose crude fiber due to their long-term life in alpine grasslands (Liu et al., 2022). We speculate that the unique rumen microflora of Tibetan sheep can better decompose and utilize high fiber agricultural byproducts such as wheat straw. The rumen microbiota composition and metabolite changes affect animal growth and meat quality. Whereas untargeted metabolomics combined with microbiomics is often used to study the rumen microbiota of living organisms and the metabolism of the host, it can express more intuitively how rumen microorganisms and their metabolites affect the metabolic activities and production performance of the host (Zhang et al., 2022b). As shown in previous studies, different feed compositions can alter the relative abundance of the rumen Bacteroidetes, Firmicutes and Proteobacteria as well as promote carbohydrate, amino acid and energy metabolism functions (Park et al., 2020; Zhao et al., 2021). High energy level feeds can provide substrates for fatty acid synthesis by increasing the relative abundance of rumen amylolytic bacteria, thus improving meat quality, such as fat content and shear force in the longissimus dorsi of yaks (Du et al., 2021). One study used the microbiome and metabolome to reveal the mechanisms by which triterpene saponins regulate rumen metabolism in Holstein cows and found that the saponins regulated rumen lipid metabolism by decreasing estradiol and isoflavones aglycone concentrations through lowering *Lachnospiraceae_NK3A20_group* abundance in the rumen (Wang et al., 2019). Overall, feed is a key factor influencing the microbial composition and function of the rumen in ruminants, which alters rumen microbiology and metabolism (Olijhoek et al., 2022). This process subsequently alters metabolite deposition in muscle and affects meat quality. We therefore focused on exploring meat quality, rumen microbiota and metabolites and correlations in Tibetan sheep fed different roughages, which are lacking in current research.

This study aims to investigate the characteristics of rumen microbiota and metabolites in Tibetan sheep in response to different types of roughages and their impact on meat quality. We used 16S rRNA sequencing and gas chromatography–mass spectrometry (GC–MS) techniques to analyze the effects of different roughage on the bacterial composition and fermentation parameters of the rumen of Tibetan sheep. We investigated rumen metabolites using ultrahigh performance liquid chromatography quadrupole time of flight mass spectrometry (UHPLC-QTOF-MS). The correlation between carcass and meat quality characteristics of the longissimus dorsi in Tibetan sheep and rumen bacteria, metabolites and rumen fermentation parameters was also analyzed. This study offers novel insights into the impact of various roughages on meat quality traits, rumen microorganisms, and metabolites in domesticated Tibetan sheep. The results will have some practical value for local herders.

## Materials and methods

### Animal management and sample collection

All animal studies were approved by the Animal Committee of Gansu Agricultural University (Approval No. GAU-LC-2020-27). Sixty 3 month old Tibetan sheep (22.3 ± 3 Kg) of similar age and healthy body condition were selected and randomly divided into three groups with 20 animals in each group, namely, the wheatgrass group (TW, Diet contains 50% wheatgrass chopped at 15 cm), whole corn silage group (TS, diet containing 50% whole corn silage) and a mixed group (TM, diet containing 25% each of whole corn silage and wheatgrass), and the nutrient composition of each feed material is shown in Supplementary Table S1. The diet composition and nutrient composition of the diets used for the experimental sheep are shown in Table 1. The feeding trial was conducted for 143 days, with a 15-day pretest period and a 128-day formal trial period. At the end of the feeding experiment, six sheep in each group were randomly selected for slaughter, and samples of the longissimus dorsi between the 12th and 13th ribs of each sheep’s left half carcass were collected; the meat quality was determined immediately on site. After slaughter, the rumen fluid was immediately filtered through four layers of sterile gauze into sterile freezing tubes and then placed in a liquid nitrogen tank and brought back to the laboratory for storage at −80°C for ruminal fermentation parameters, 16S rRNA sequencing and metabolomics assays.

**Table 1:** Compositions and nutrient levels of diets (DM basis).

Items		TW	TM	TS
Content (%)	Whole corn silage	0.00	25.00	50.00
	Wheat straw	50.00	25.00	0.00
	Corn	22.50	20.40	17.30
	Wheat bran	1.00	8.00	16.00
	Bean pulp	9.00	10.00	8.20
	Cottonseed meal	12.00	6.10	3.00
	Calcium carbonate	0.50	0.50	0.50
	Bicarb	0.50	0.50	0.50
	Salt	0.50	0.50	0.50
	4% Premix^a^	4.00	4.00	4.00
Nutrient	Dry matter, DM (%)	91.39	76.37	61.40
	Digestible energy, DE (M J kg^−1^)	10.86	11.36	11.97
	Crude Protein, CP (%)	13.27	13.23	13.22
	Ether extract, EE (%)	2.33	2.66	3.19
	Coarse ash, Ash (%)	7.22	7.56	7.96
	Neutral detergent fiber, NDF (%)	41.46	34.06	27.19
	Acid detergent fiber, ADF (%)	29.03	23.49	18.20
	Calcium, Ca (%)	1.09	1.11	1.13
	Phosphorus, P (%)	0.46	0.48	0.45

a One kilogram of the premix contained the following: VA 450000 IU, VD2 100,000 IU, VE 150000 IU, Fe 350 mg, Cu 3,500 mg, Mn 600 mg, Zn 350 mg, Co 10 mg, Se 20 mg. Digestible energy was calculated, and other values were measured. TW: wheatgrass group; TS: Whole corn silage group; TM: Mixed group. The same below.

### Carcass and meat quality determination

The longissimus dorsi was vertically printed on sulfuric acid paper and then the eye muscle area was calculated using square paper. Back fat thickness was measured with vernier calipers. Measurement of a* (redness), b* (yellowness) and L* (brightness) values of mutton using a colorimeter (CR-10 plus, Konica Minolta, Japan). Direct determination of the pH of meat using a pH meter (Testo 205, Testo, Germany). The mutton was sampled along the muscle fiber direction and weighed (W1), then hanging the meat strips in plastic bottles at 4°C for 24 h. The weight of the meat strips was measured (W2) and the drip loss was calculated according to the following equation. $\mathrm{Drip loss}\;\left(\%\right)=\left[\left(W_{1}-W_{2}\right)/W_{1}\right]\times 100\;$ . At 45 min after slaughter, the mutton samples were scored for marbling according to the agricultural industry standard of the People’s Republic of China (NY/T 630–2002 “Evaluation and grading of lamb and mutton”). Mutton shear force with reference to Wang’s method (Wang et al., 2023). Simply put, a thermometer was inserted in the center of the mutton and then wrapped in a polyethylene bag for water bath heating; when the thermometer reached 70°C, the mutton was removed and cooled at room temperature, and when the temperature dropped to 35°C, the tenderness of the mutton was determined using a shear force meter perpendicular to the direction of the muscle fibers. In addition, the mutton samples were steamed in a water bath at 85°C for 30 min to calculate the cooking loss and cooked meat percentage.

### Rumen fermentation characteristics

Ruminal fluid pH was measured using a pH-3c acidity meter immediately after slaughter; The phenol sodium hypochlorite colorimetric method was used to determine the concentration of NH_3_-N (Wang et al., 2009); Volatile fatty acids (VFA) were determined using gas chromatography according to the previous procedure (GC-2010 Plus; Shimadzu, Kyoto, Japan) (Wang Z. et al., 2022).

### Rumen microbial diversity analysis

Ruminal microbiome DNA was extracted from rumen fluid samples using a bacterial DNA extraction kit (Omega, Shanghai, China) and then tested for DNA purity and concentration using agarose gel electrophoresis and Thermo NanoDrop 2000 ultra-microscopic spectrophotometer (Thermo Fisher Scientific, Waltham, MA, USA), respectively. PCR amplification of the V3-V4 region of the 16S rRNA gene was performed using universal primers 338\F (5′-ACTCCTAC GGGAGGCAGCAG-3′) and 806 R (5′-GGACTACHVGGGTWTCTAAT-3′) after passing the quality control. The amplification procedure was: predenaturation at 95°C for 3 min, denaturation at 95°C for 30 s, annealing at 55°C for 30 s, extension at 72°C for 30 s, and 40 cycles. Finally, the amplification products were sequenced and analyzed on the Illumina MiSeq (Illumina, San Diego, CA, United States) platform. After sequencing, the raw data were first quality filtered using Trimmomatic (version 0.33). Primer sequence removal using Cutadapt (version 1.9.1), USEARCH (version 10) and UCHIME (version 8.1), double ended reads for the final high quality sequences were obtained for subsequent analysis by splicing and removing chimeras. Sequences were clustered at a 97% similarity level using USEARCH software, and species annotation was performed by comparison with Silva’s (Release 138, http://www.arbsilva.de) database to analyze each sample’s microbial community structure and species clustering. The alpha diversity index was calculated and sample dilution and rank abundance curves were plotted using QIIME2 software. Beta diversity analysis was used to assess the variation in sample colony composition and structure, and sample heat map clustering and principal coordinate analysis (PCoA) were plotted based on the R language platform. Anosim and Adonis tested differences between groups to see if they were significantly greater than differences within groups. Line discriminant analysis (LDA) effect size (LefSe)^1^ was analyzed for between group differences biomarkers. Species abundance data were compared between groups using t-tests in Metastats^2^ software to screen for species that differed between the two sample groups.

### Untargeted metabolomics analysis of rumen contents

Accurately extract 100 μL of rumen contents, add 500 μL of extraction solution containing internal standard (methanol: acetonitrile volume ratio = 1:1, the internal standard is 2-Chloro-L-phenylalanine, concentration 20 mg/L), vortex and mix for 30 s, then sonicate for 10 min (ice water bath) and leave for 1 hour (−20°C); The samples were then centrifuged for 15 min (4°C, 12000 rpm); The extract was dried and concentrated by removing 500 μL of supernatant, followed by the addition of 160 μL of acetonitrile to water at a volume ratio of 1:1; Repeat the previous step of vortexing, sonication and centrifugation and then remove the supernatant. Determination by Waters Xevo Acquity I Class PLUS ultra performance liquid chromatography tandem with a Waters Xevo Xevo G2-XS QTOF high resolution mass spectrometer. The chromatographic column was a Waters Xevo Acquity UPLC HSS T3 (1.8um 2.1*100 mm). The mobile phase A is 0.1% formic acid aqueous solution and mobile phase B is 0.1% formic acid acetonitrile in positive ion mode (POS); The mobile phase A in negative ion mode (NEG) was 0.1% formic acid aqueous solution and mobile phase B: 0.1% formic acid acetonitrile. MassLynx (V4.2) software was used for primary and secondary mass spectrometry data acquisition in MSe mode with a low collision energy of 2 V and a high collision energy interval of 10–40 V. The scan frequency was 0.2 s for one mass spectrometer. Data processing operations such as peak extraction and peak alignment were done using Progenesis QI software, and identification was carried out based on the METLIN database and public database. In contrast, theoretical fragmentation identification was carried out with the deviation of parent ion mass number within 100 ppm and fragmentation ion mass number within 50 ppm. Bioinformatics analysis of metabolites was performed using BMKCloud,^3^ and principal component analysis and Spearman correlation analysis was used to determine the reproducibility of samples within groups and quality control samples. The taxonomic and pathway information of the identified compounds was searched in the KEGG (Kyoto Encyclopedia of Genes and Genomes), HMDB (Human Metabolome Database) and lipidmap (Lipid Metabolites and Pathways Strategy) databases. According to the grouping information, the multiplicity of differences was calculated, and T-test calculated the *p*-value of the different significance of each compound. OPLS-DA modeling using the R language package ropls. Differential metabolites were screened using a combination of difference multiples, *P* and variables important in the projection (VIP) value of the OPLS-DA model. The screening criteria were *p* < 0.05 and VIP > 1. Calculate the KEGG pathway with significant enrichment of differential metabolites using a hypergeometric distribution test (Yu et al., 2012).

### Data analysis

The Kolmogorov–Smirnov and Leven test procedures of SPSS (SPSS v 26.0, SPSS, Inc., Chicago, Illinois, United States) were used to check the data’s normality and homogeneity of variance before performing statistical analyses. One-way ANOVA was performed using SPSS software, and Duncan’s method was used for multiple comparisons when differences were significant, and *p* < 0.05 was considered significant. Spearman’s correlation coefficient was used to test the relationship between rumen bacterial composition, metabolites and carcass characteristics and meat quality characteristics in Tibetan sheep, with significant correlations of *p* < 0.05 and R > 0.60.

## Results

### Carcass and meat quality characteristics

As shown in Table 2, the average daily gain (ADG), body weight before slaughter, carcass weight, dressing percentage, area of longissimus dorsi, back fat thickness and intramuscular fat (IMF) content were significantly higher in the TM and TW groups than in the TS group (*p* < 0.05). The marble pattern was significantly higher in the TW group than in the TM and TS groups (*p* < 0.05). The L* values in the TW and TS groups were significantly higher than those in the TM group (*p* < 0.05). The TS group had significantly lower a* values than the other two groups (*p* < 0.05). The TW group’s shear force and cooking loss were significantly lower than the other two groups (*p* < 0.05). The pH, fat color and drip loss were significantly higher in the TS group than in the TW and TM groups (*p* < 0.05).

**Table 2:** Analysis of carcass and meat quality characteristics.

Item			TW	TS	TM	*p-*value
Carcass characteristics	ADG(g)		279.33 ± 9.62^b^	267.83 ± 4.21^c^	310.33 ± 11.30^a^	<0.001
	Body weight before slaughter(kg)		64.60 ± 1.54^b^	59.33 ± 1.03^c^	67.60 ± 0.66^a^	<0.001
	Carcass weight (kg)		30.37 ± 0.58^b^	24.50 ± 3.87^c^	33.80 ± 1.03^a^	<0.001
	Dressing percentage(%)		47.44 ± 1.75a	41.22 ± 5.85^b^	49.77 ± 1.47^a^	0.003
	Area of longissimus dorsi(cm^2^)		25.11 ± 1.27^a^	19.45 ± 1.21^c^	22.67 ± 2.18^b^	<0.001
	Back fat thickness(mm)		11.67 ± 1.51^a^	8.45 ± 0.69^b^	13.22 ± 1.86^a^	<0.001
Meat quality characteristics	PH_45min_		6.47 ± 0.08ab	6.50 ± 0.07^a^	6.36 ± 0.14^b^	0.091
	PH_24h_		5.93 ± 0.04^b^	6.09 ± 0.07^a^	6.06 ± 0.04^a^	<0.001
	Marble pattern		3.50 ± 0.32^a^	2.75 ± 0.27^c^	3.12 ± 0.40^b^	0.001
	Meat color	L	25.89 ± 0.85^a^	27.00 ± 0.92^a^	24.13 ± 1.23^b^	0.001
		a	18.13 ± 0.93^a^	15.43 ± 0.55^b^	17.63 ± 1.31^a^	0.001
		b	4.92 ± 0.94	5.22 ± 0.61	4.65 ± 0.62	0.434
	Intramuscular fat (%)		3.40 + 0.21^a^	1.94 + 0.08^c^	2.48 + 0.18^b^	<0.001
	Shear force (N)		83.05 ± 3.22^c^	90.10 ± 4.62^b^	97.27 ± 6.56^a^	0.001
	Drip loss (%)		4.42 ± 0.33^b^	5.46 ± 0.24^a^	4.22 ± 0.42^b^	<0.001
	Cooking loss (%)		12.03 ± 1.09^b^	14.69 ± 2.29^a^	14.25 ± 2.53^a^	0.049
	Cooking percentage (%)		66.45 ± 2.74	61.94 ± 6.03	65.11 ± 1.74	0.163
	Fat color		2.33 ± 0.52^b^	3.33 ± 0.52^a^	2.66 ± 0.52^b^	0.013

TW: wheatgrass group; TS: Whole corn silage group; TM: Mixed group. a, b, c Values with different superscripts in the same row are significantly different (*P* < 0.05), The same below.

### Rumen fermentation characteristics

As shown in Table 3, the NH_3_-N content, TVFA, and propionate content of the TM and TW groups were significantly higher than those of the TS group (*p* < 0.05). The pH of the TW group was significantly higher than that of the TS group (*p* < 0.05). The butyrate content and A/P were significantly higher in the TS group than in the other two groups (*p* < 0.05). The butyrate, isovaleric acid content and A/*p* values were significantly higher in the TS group than in the other two groups (*p* < 0.05).

**Table 3:** Analysis of rumen fermentation parameters.

Items	TW	TS	TM	*p-*value
Acetate (%)	61.42 ± 2.36	59.80 ± 3.52	58.54 ± 1.71	0.199
Propionate (%)	25.12 ± 4.89^a^	20.41 ± 4.18^b^	26.71 ± 3.80^a^	0.048
Isobutyric acid (%)	0.52 ± 0.04^b^	0.81 ± 0.11^b^	2.13 ± 1.48^a^	0.012
Butyrate (%)	11.10 ± 1.50^b^	15.76 ± 1.03^a^	12.44 ± 1.21^b^	0.042
Isovaleric acid (%)	1.19 ± 0.30^b^	2.25 ± 0.55^a^	0.98 ± 0.28^b^	<0.001
Valerianic acid (%)	0.65 ± 0.04	0.71 ± 0.05	0.77 ± 0.06	0.008
TVFA (mmol l^−1^)	97.54 ± 1.89^a^	84.57 ± 4.65^b^	99.42 ± 3.84^a^	0.018
A/P	2.66 ± 0.51^b^	3.20 ± 0.79^a^	2.29 ± 0.32^b^	0.045
pH	6.04 ± 0.08^a^	5.91 ± 0.12^b^	5.98 ± 0.15^ab^	0.040
NH_3_-N (mg dL^−1^)	4.86 ± 0.15^a^	3.93 ± 0.17^b^	5.10 ± 0.28^a^	0.003

TVFA: Total volatile fatty acids. A/P: the ratio of acetic acid to propionic acid. The unit % represents the proportion of a single VFA in the TVFA.

^a, b, c^Values with different superscripts in the same row are significantly different (*p* < 0.05).

### Rumen microbiota composition

**Figure 1:**
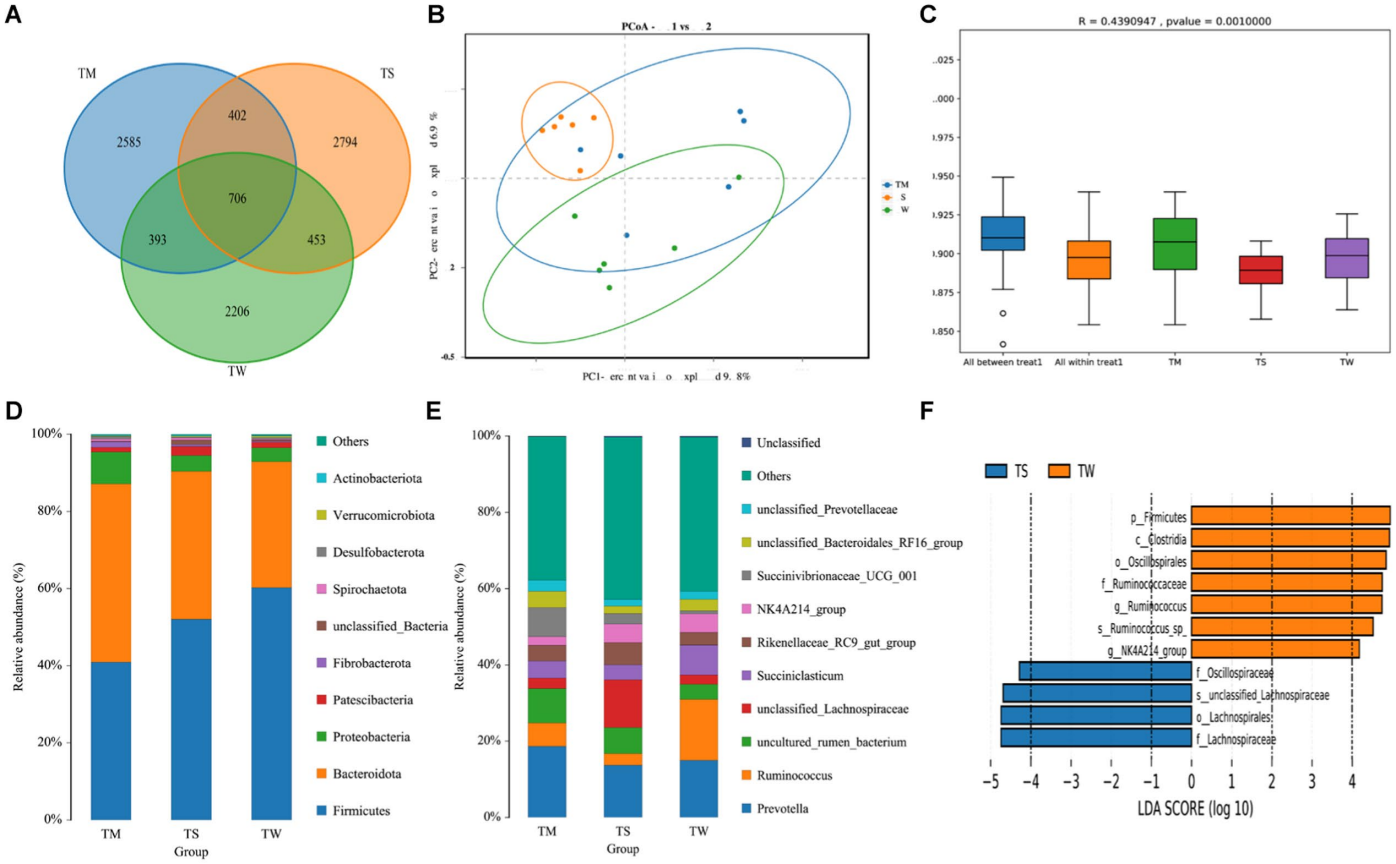
**(A)** OTU-Venn analysis; **(B)** PCoA plot; **(C)** Anosim analysis; **(D,E)** Relative abundance of phylum and genus horizontal species; **(F)** Significantly different bacterial taxa identified by the linear discriminant analysis effect size (LEfSe).

A total of 1,439,771 Reads were obtained for the three groups of 18 samples, and 1,436,937 Clean Reads were obtained after quality control and splicing, yielding an average of 79,830 Clean Reads per sample. There were 2,206, 2,585 and 2,794 OUTs unique to the TW, TM and TS groups, respectively, and 706 OUTs common to all three groups (Figure 1A). The α-diversity of the three groups was not significantly different (Supplementary Table S2). Dilution curves show a plateau at 20,000 reads, indicating saturation of sequencing coverage; Species accumulation plots show that the number of species and shared species in the environment saturates with increasing sample size (Supplementary Figure S1). PCoA shows a good separation of samples in each group (Figure 1B); Anosim analysis further demonstrated significant differences between groups (Figure 1C). We further identified 18 phyla, 25 classes, 32 orders, 62 familys, 164 genus and 194 species of microorganisms taxonomically. At the phylum level, the relative abundance of Bacteroidota was higher and that of Firmicutes was lower in the TM group compared to the TW group (*p* < 0.01) (Figure 1D; Supplementary Table S3). At the genus level (Figure 1E; Supplementary Table S3), the *Prevotella*, *Ruminococcus* and *Uncultured_rumen_Bacterium* were the dominant genus in the three groups. Among the top 10 genus in terms of abundance, the relative abundance of *Rikenellaceae_RC9_gut_group* was significantly higher in the TS group than in the TW group (*p* < 0.01). While the relative abundance of *Succiniclasticum*, *Ruminococcus* and *Candidatus_Saccharimonas* in the TW group was significantly higher than that in the TS group (*p* < 0.01). The relative abundance of *Prevotella_7* was higher in the TM group compared to the other two groups (*p* < 0.01). We also found that *Alloprevotella* and *Lachnospiraceae_NK3A20* were significantly higher in the TM group than in the TS group (*p* < 0.01). LEfSe of samples between groups showed 7 and 4 significantly different biomarkers in the TW and TS groups, respectively (LDA score > 4) (Figure 1F).

### Rumen metabolomic profiles

**Figure 2:**
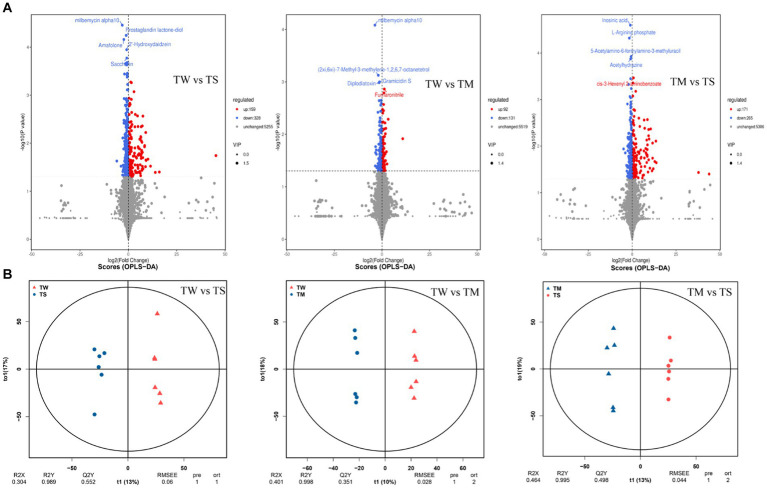
**(A)** Volcanic maps of differential metabolites in the three groups; **(B)** The OPLS-DA model score plots in the three groups.

**Figure 3:**
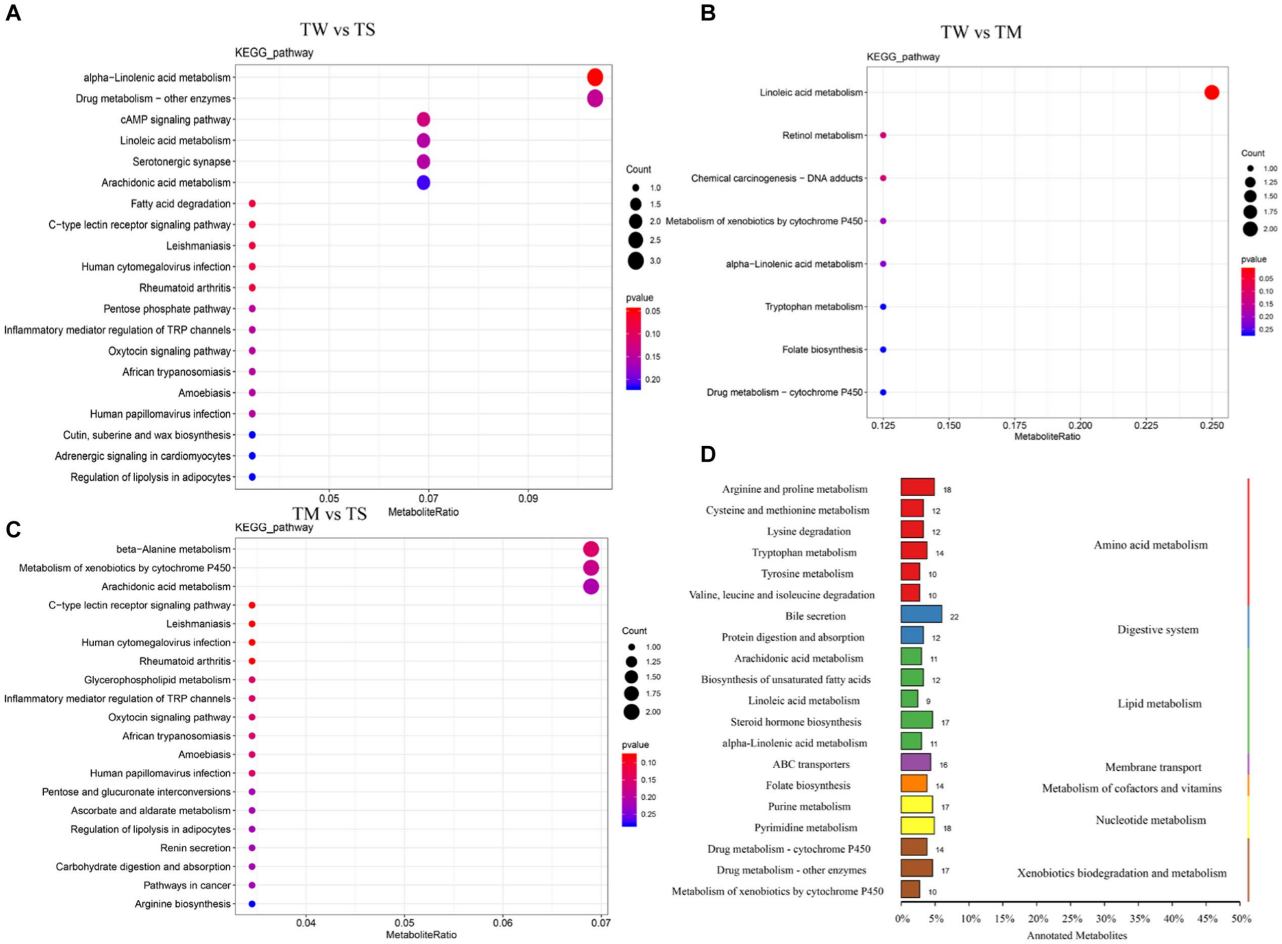
**(A–C)** KEGG map of rich spots; **(D)** Classification diagram of differential metabolites.

It can be seen from the OPLS-DA model that R2Y is close to 1 for all comparisons between groups (Figure 2B), and the slope of the fitted regression line for Q2Y is positive (Supplementary Figure S2), indicating that the established model is stable and reliable and can be used to compare the differences between the two groups. Analysis with *p* < 0.05 and VIP > 1 as screening criteria revealed that 487, 223 and 436 differential metabolites were identified between the TW vs. TS, TW vs. TM and TM vs. TS groups, respectively (Figure 2A). The PCA clustering plot showed that the metabolic profiles of the TS group were more different from those of the TW and TM groups, respectively, while the metabolic profiles of the TM and TW groups were less different (Supplementary Figure S3). The TW group had significantly higher contents of beta-alanyl-L-lysine, prostaglandin E2, stearidonic acid, N-acetyl-L-glutamate 5-phosphate, glutaric acid, 9R,10S-epome, dihomo-gamma-linolenate, and D-ribose were significantly higher than those in the TS group. At the same time, 6-hydroxymelatonin, indoxyl, 5-phosphonooxy-L-lysine, and 5,6-DHET were significantly lower than those in the TS group. The contents of CDP-ethanolamine, dCDP, 5-methylcytosine, docetaxel, prostaglandin E2, beta-alanyl-L-lysine, prostaglandin E2, and pyridoxine phosphate in the TM group were significantly higher than the TS group (Supplementary Table S4). These critical differential metabolisms mainly participated in amino acid metabolism (76), digestive system (34) and lipid metabolism (60) pathways (Figure 3D; Supplementary Table S5).

### Metabolic pathways of differential metabolites

Differential metabolite KEEG enrichment analysis is illustrated in Figures 3A–C. The critical differential metabolic pathways between the TW group and the TS group were alpha-linolenic acid metabolism, tryptophan metabolism, arachidonic acid metabolism, linoleic acid metabolism, cAMP signaling pathway, tyrosine metabolism and biosynthesis of unsaturated fatty acids. The critical differential metabolic pathways between the TM and TS groups were arachidonic acid metabolism, bile secretion, beta-alanine metabolism and cAMP signaling pathway (Supplementary Table S6).

### Microbiome-metabolome-phenotypic index joint analysis

**Figure 4:**
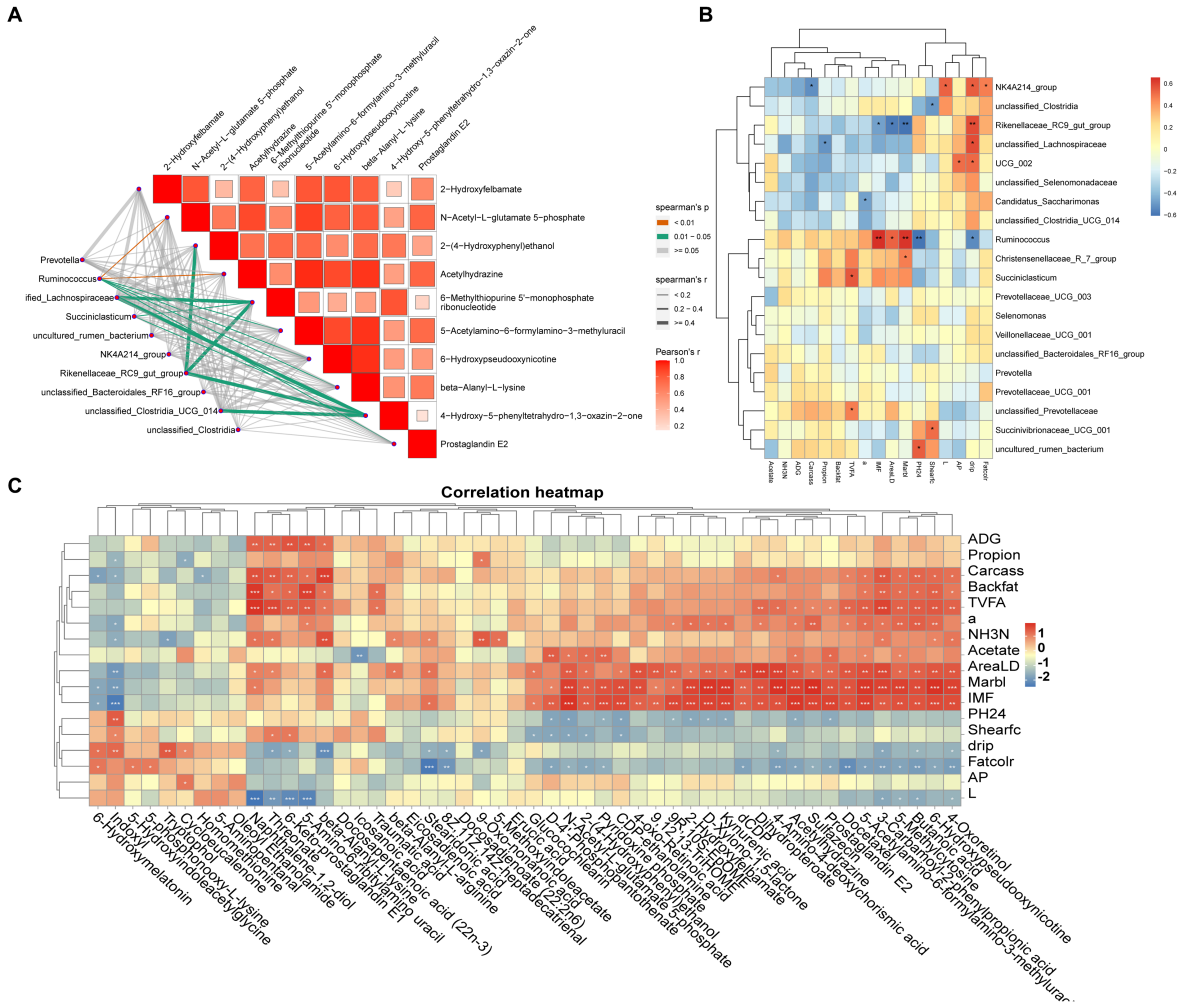
**(A)** Microbiome-Metabolome network heat map; **(B)** Microbiome-Phenotypic index correlation heat map; **(C)** Metabolome-Phenotypic correlation heat map.

Spearman’s correlation coefficient model analyzed the correlation between genuslevel differential microorganisms and differential metabolites (*p* < 0.05 and R > 0.60) and revealed that *Ruminococcus*, *Succiniclasticum*, *Rikenellaceae_RC9_gut_group*, *Succinivibrionaceae_UCG_001* and metabolites such as beta-alanyl-L-lysine, prostaglandin E2, and N-acetyl-L-glutamate-5-phosphate were significantly correlated (*p* < 0.05) (Figure 4A). The association between differential microorganisms and meat quality was also found (Figure 4B), such as *Ruminococcus* and *Succiniclasticum* were significantly positively correlated with IMF, eye muscle area and L* value (*p* < 0.05), while the *Rikenellaceae_RC9_gut_group* and *NK4A214_group* were significantly and positively correlated with drip loss(*p* < 0.05). Further analysis of rumen differential metabolites with meat quality and rumen fermentation parameters revealed that metabolites such as stearidonic acid, 6-hydroxypseudooxynicotine, icosanoic acid, beta-alanyl-L-lysine, prostaglandin E2 and indoxyl were significantly and positively correlated with TVFA and propionic acid as well as with meat traits such as ADG, IMF and shear force (*p* < 0.05) (Figure 4C).

## Discussion

This study investigated Tibetan sheep’s growth performance and meat quality indexes fed different roughage diets and further analyzed rumen fermentation parameters, rumen microbiome and metabolomics to extend the rumen microbial perspective of Tibetan sheep on different roughage utilization. Previous studies have found that adding wheat straw to sheep and cow diets reduces feed intake and performance (Wang et al., 2014; Mahmoudi-Abyane et al., 2020). We came to a different conclusion and found that feeding wheat straw to Tibetan sheep increased growth performance (body weight before slaughter, carcass weight), Carcass characteristics (dressing percentage, area of longissimus dorsi and back fat thickness) and modified meat quality (marble pattern, L*, IMF, shear force and cooking loss). We found that rumen fluid pH was significantly higher in the added wheat straw than in the whole corn silage group, and some studies have shown that wheat straw increases rumen pH affecting rumen fibrolytic bacteria to increase fiber digestibility (Kahyani et al., 2019). In contrast, whole corn silage resulted in lower rumen pH (Sulzberger et al., 2016), which may explain the lower production performance of Tibetan sheep in the TS group. The better growth performance and carcass characteristics of wheat straw fed Tibetan sheep in this study were attributed to the higher total VFA, acetate and propionic acid content in the rumen, as the high propionic acid content and the ratio of acetate to propionic acid (A:P) implied higher energy utilization (Poudel et al., 2019). Meat color is an essential indicator of meat characteristics. The Tibetan sheep feeding wheat straw had higher a* and lower L* and b*, suggesting that feeding wheat straw improves meat color (Hughes et al., 2020). A study has shown that lower muscle drip loss leads to lower L* due to a lower refractive index of the muscle surface; lower drip loss and cooking loss in the wheat straw group in the present study were positively correlated with low L* suggesting that feeding wheat straw improves the water holding capacity of the mutton (Kim et al., 2011). The water holding capacity of the muscle improves as the muscle pH decreases and the intramuscular fat content increases (Moreno et al., 2020). Thus, feeding wheat straw may improve muscle water holding capacity by slowing the rate of muscle pH decline and increasing fat deposition, thereby reducing L* values.

It has been found that changes in rumen microbiota significantly impact the formation of meat quality traits in ruminants (Zhang et al., 2022b). Therefore, we performed 16S rRNA sequencing, and the PCoA and Anosim analyses revealed that different roughage altered the rumen microbial community in Tibetan sheep. The bacteria at the genus level in the three groups of rumen contents samples in this study were mainly *Prevotella* and *Ruminococcus*, which is consistent with previous studies (Xue et al., 2017). The *Prevotella* abundance was highest in the TM group, and *Prevotella* effectively degraded hemicellulose and starch. In addition, *Prevotella* is thought to be associated with propionic acid production (Lv et al., 2019). *Alloprevotella* was found to be closely related to VFA production and may play an important role in the fermentation of structural carbohydrates in the rumen of Tibetan sheep, thus promoting energy absorption (Fan et al., 2020). *Succiniclasticum* has also been reported to convert succinic acid into propionic acid (van Gylswyk, 1995). High dietary fiber content steadily increases *Ruminococcus* abundance and promotes fiber fermentation into short-chain fatty acids (Tomova et al., 2019). Feeding Tibetan sheep with high-fiber wheat straw promotes the relative abundance of *Succiniclasticum* and *Ruminococcus* (Zhang et al., 2018), increasing VFA and propionic acid content within the TM group. The elevated VFA and propionic acid levels provide ample energy for Tibetan sheep (Nathani et al., 2015), ultimately enhancing their production performance in the TW group.

Ruminal microorganisms are known as the “second genome,” The microbiota affects a wide range of host physiological functions and meat quality through metabolites (Ren et al., 2018; Wang et al., 2021). We further analyzed the metabolic functions of the rumen microbiota under different roughage feeds using UHPLC-QTOF-MS. Previous studies have reported that some rumen bacteria are associated with the deposition of AA and lipid metabolites in muscle (Wang et al., 2021). Similar results were observed in our study, which identified many differential metabolites mainly involved in amino acid metabolism, digestive system and lipid metabolism. The differential metabolites in the TW and TM groups compared to the TS group were significantly enriched in alpha-linolenic acid metabolism, arachidonic acid metabolism, tryptophan metabolism, linoleic acid metabolism and cAMP signaling pathways. These metabolic pathways are closely related to energy utilization and meat quality traits. Arachidonic acid is an abundant unsaturated fatty acid that can be used as a specific plasma marker for daily weight gain in bulls (Artegoitia et al., 2017). In addition, high levels of arachidonic acid were better to activate PPAR-α to increase IMF (Feng et al., 2018). alpha-linolenic acid is an Omega-3 polyunsaturated fatty acid (Shahidi and Ambigaipalan, 2018), and alpha-linolenic acid and linoleic acid have been associated with amino acid and fatty acid deposition in muscle (Wang B. et al., 2020). In this study, some metabolites enriched in arachidonic acid, alpha-linolenic acid and linoleic acid signaling pathways were found to be significantly elevated in Tibetan sheep after feeding on wheat straw, which suggests that Tibetan sheep can utilize essential fatty acids, such as arachidonic acid, linoleic acid and α-linolenic acid, to improve energy utilization and enhance production performance (Liu et al., 2022). As an essential aromatic amino acid, tryptophan is a biosynthetic precursor for many microbial and host metabolites (Agus et al., 2018). It has been found that high dietary protein can regulate nutrient absorption and growth performance of Tibetan sheep through the tryptophan metabolic pathway (Wang X. et al., 2022). This shows that different diets affect the growth and performance of Tibetan sheep by promoting the amino acids and lipids metabolism in the rumen. Further analysis of the critical metabolites enriched by these pathways revealed that metabolites such as beta-alanyl-L-lysine, prostaglandin E2, kynurenic acid, N-acetyl-L-glutamate 5-phosphate and D-ribose were found to be higher in the TW and TM groups. This study showed a significant positive correlation between *Ruminococcus* and N-acetyl-L-glutamate-5-phosphate and acetic acid. The N-acetyl-L-glutamate-5-phosphate is a precursor of arginine synthesis. Rumen microorganisms can use VFA or NH_3_-N as a nitrogen source for *de novo* amino acid synthesis (Kajikawa et al., 2002). This suggests that higher concentrations of acetic acid and NH_3_-N provide more substrates for amino acid synthesis in the rumen. The high concentration of organic acids, particularly kynurenic acid, in the TM group facilitates nutritional absorption and enhances growth performance in Tibetan sheep (Wang X. et al., 2022). Prostaglandin E2 plays an important role in fatty acid composition and metabolism in the liver (Stawarska et al., 2020). Previous studies reported that D-ribose induced hepatocyte lipid droplet production (Chen et al., 2019). These results may indicate that feeding wheat straw promotes fatty acid and amino acid metabolism in Tibetan sheep through rumen microbes and metabolites. This study also observed an increase in PGE1, 6-hydroxymelatonin and indoxyl levels under whole corn silage feeding. It was previously reported that degradation of carbohydrates by starch degrading bacteria such as *NK4A214_group* caused changes in 6-hydroxymelatonin and indoxyl content in the rumen (Yi et al., 2022); this also suggests that the higher abundance of *NK4A214_group* in the TS group was to adapt to high carbohydrate rations. PGE1 accumulation is associated with rumen acidosis (Mu et al., 2022), and the tryptophan metabolite (6-hydroxymelatonin, indoxyl) may maintain normal rumen pH under a high protein diet by modulating the mucosal immune system and specific receptors (Wang X. et al., 2022). This shows that the pH decrease caused by corn silage is closely related to changes in rumen microbial activity and metabolites. Besides, we also identified many differential metabolites, such as D-xylono-L,5-lactone, threonate, butanoic acid, icosanoic acid, eicosadienoic acid, (9Z)-hexadecenoic acid and CDP-ethanolamine, which are all involved in meat quality regulation. In conclusion, the significant differences in rumen microbes and metabolites among the three groups reflect the characteristics of rumen microbiota and metabolites in Tibetan sheep in response to different types of roughage and their effects on meat quality. However more in-depth mechanisms need to be studied further.

## Conclusion

This study demonstrated that the inclusion of wheatgrass in the diet of Tibetan sheep increased the abundance of bacteria such as *Ruminococcus* and *Succiniclasticum*, as well as amino acid and lipid metabolites including N-acetyl-L-glutamate-5-phosphate, D-ribose, and prostaglandin E2. These changes ultimately led to improved production performance, such as ADG and IMF, enhancing meat quality like shear force and meat color. Specifically, The diet containing 25% each of whole corn silage and wheatgrass was optimal for promoting superior performance outcomes among Tibetan sheep.

## Data availability statement

The datasets presented in this study can be found in online repositories. The names of the repository/repositories and accession number(s) can be found in the article/Supplementary material.

## Ethics statement

The animal study was approved by Animal Committee of Gansu Agricultural University. The study was conducted in accordance with the local legislation and institutional requirements.

## Author contributions

YR, ML, and ZW designed the study. YR, YZ, and ZW performed the experiments and collected the samples. YR and ZW analyzed the data and wrote the manuscript. All authors contributed to the article and approved the submitted version.

## Conflict of interest

The authors declare that the research was conducted in the absence of any commercial or financial relationships that could be construed as a potential conflict of interest.

## Publisher’s note

All claims expressed in this article are solely those of the authors and do not necessarily represent those of their affiliated organizations, or those of the publisher, the editors and the reviewers. Any product that may be evaluated in this article, or claim that may be made by its manufacturer, is not guaranteed or endorsed by the publisher.

## Supplementary material

The Supplementary material for this article can be found online at: https://www.frontiersin.org/articles/10.3389/fmicb.2023.1247609/full#supplementary-material

## Footnotes

^1^ http://huttenhower.sph.harvard.edu/lefse/

^2^ http://metastats.cbcb.umd.edu/

^3^ www.biocloud.net
