# Supplementary material for: Responses of rumen microorganisms and metabolites to different roughage of domesticated Tibetan sheep
Source: Front Microbiol. 2023 Aug 17;14:1247609. doi: 10.3389/fmicb.2023.1247609 (PMC10469951; doi:10.3389/fmicb.2023.1247609)
Supplement: Supplementary file 1 [file Data_Sheet_1.zip › Tables S1-S2 and Figures S1-S2.pdf]

TABLE S1| Nutrient composition of feed materials (DM basis)

Items	Corn	Wheat bran	Bean pulp	Cottonseed meal	Whole corn	Wheat straw
Dry matter, DM (%)	87.92	88.68	89	90.95	33.41	93.07
Crude Protein, CP (%)	8.67	16.9	44.1	43.09	8.27	3.76
Ether extract, EE (%)	3.07	3.23	1.9	1.78	4.84	0.96
Coarse ash, Ash (%)	1.17	4.18	6.1	6.54	6.35	4.73
Neutral detergent fiber, NDF (%)	10.67	14.44	13.6	30.68	42.01	68
Acid detergent fiber, ADF (%)	7.61	8.77	9.6	18.08	28.31	48.4
Calcium, Ca (%)	0.08	0.03	0.33	0.23	0.23	0.11
Phosphorus, P (%)	0.17	0.2	0.62	0.93	0.09	0.23

TABLE S2| Effects of different roughage treatments on diversity index

Index Type	TM	TS	TW	SEM	*p*
Shannon	7.7721	7.7844	7.6493	0.16594	0.771
Simpson	0.988	0.9602	0.9857	0.01024	0.318
ACE	938.7133	1041.5697	876.671	35.74079	0.077
Chao1	931.3028	1034.8675	870.804	35.66539	0.078

TW: wheatgrass group; TS: Whole corn silage group; TM: Mixed group. SEM: Standard error mean, The same below.

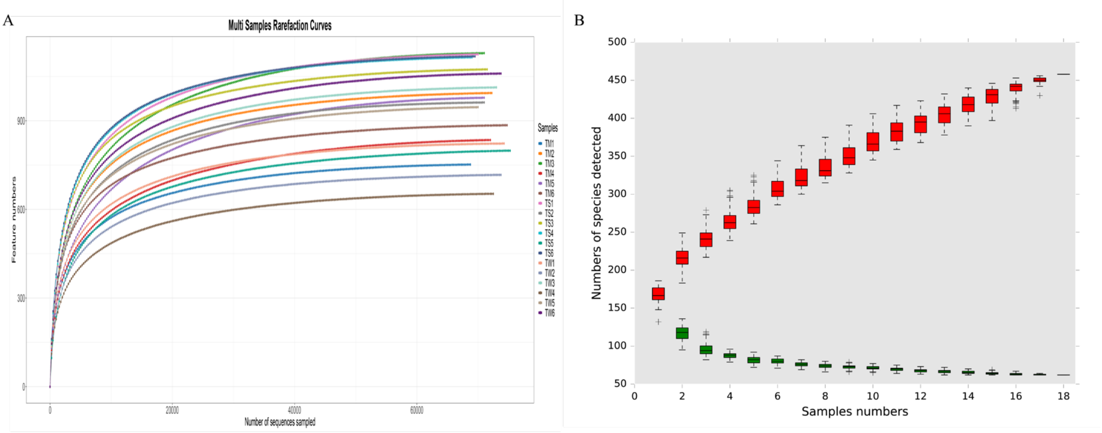

FIGURE S1 | Diversity analysis. (A) dilution curve analysis; (B) Species accumulation curve.

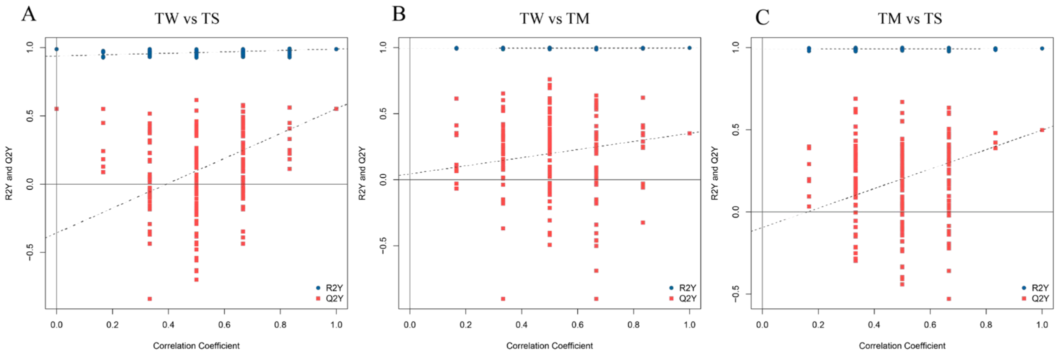

FIGURE S2 | OPLS-DA model replacement test diagram. If the slope of Q2Y fitting regression line is regular, it means that the model is meaningful, and the blue points are generally above the red points, it means that the independence of the modeling training set and the test set is good.

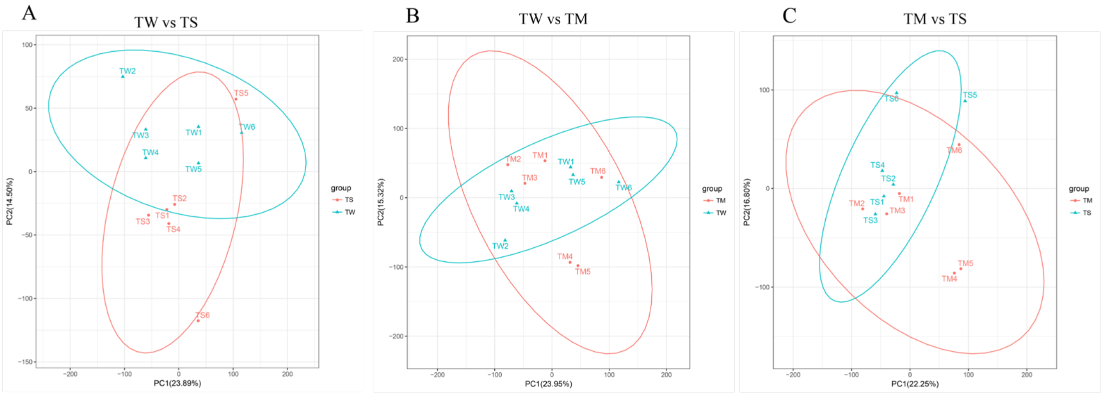

FIGURE S3 | Principal Component Analysis.
